# A latent class analysis of dietary behaviours associated with metabolic syndrome: a retrospective observational cross-sectional study

**DOI:** 10.1186/s12937-020-00636-7

**Published:** 2020-10-16

**Authors:** Jung Ha Park, Ju Young Kim, So Hye Kim, Jung Hyun Kim, Young Mi Park, Hye Seon Yeom

**Affiliations:** 1 Department of Family Medicine, Jeju National University Hospital, Jeju, South Korea; 2 Department of Family Medicine, Seoul National University Bundang Hospital and Seoul National University College of Medicine, 82, Gumi-ro 173 Beon-gil, Bundang-gu, Seongnam, Gyeonggi-do 13620 Republic of Korea; 3 Nutrition Care Services, Seoul National University Bundang Hospital, Seongnam, South Korea; 4 Department of Psychiatry, Seoul National University Bundang Hospital, Seongnam, South Korea

**Keywords:** Dietary behaviour, Eating behaviour, Emotional eating, Mealtimes, Meal frequency, Obesity, Metabolic syndrome, Latent class analysis

## Abstract

**Background:**

Obesity defined solely by the Body Mass Index (BMI) may not reflect the true heterogeneity of the obese population. This study aimed to classify the dietary behaviours of overweight and obese individuals and to explore the relationship between patterns of dietary behaviour and cardiometabolic risk factors.

**Methods:**

A total of 259 patients who visited an outpatient weight management clinic at a tertiary hospital and underwent a dietary behaviour assessment between January 2014 and February 2019 were enrolled in the study. Dietary behaviours were assessed in three domains with nine categories, including choice of food (frequently eating out and consumption of instant/fast/takeaway food), eating behaviour (irregular meals; frequent snacking, including eating at night; emotional eating; and overeating/binge eating), and nutrient intake (high-fat/high-calorie foods, salty food, and poorly balanced diet). Latent class analysis (LCA) was used to classify the subjects according to these categories. Associations between latent class and metabolic syndrome were assessed by logistic regression.

**Results:**

The subjects were classified into three LCA-driven classes, including a referent class of healthy but unbalanced eaters (*n* = 118), a class of emotional eaters (*n* = 53), and a class of irregular unhealthy eaters (*n* = 88). Compared with the referent class, emotional eaters had a significantly higher BMI (beta = 3.40, *P* < 0.001) accompanied by metabolic syndrome (odds ratio 2.88, 95% confidence interval 1.16–7.13).

**Conclusions:**

Our three LCA-driven obesity phenotypes could be useful for assessment and management of obesity and metabolic syndrome. The association between emotional eaters and higher BMI and metabolic syndrome was stronger than that with other eaters. Thus, emotional regulation strategies might have benefit for emotional eater’s diet.

## Background

The World Health Organization has declared obesity to be a global epidemic with a prevalence that has tripled since 1975 and complications that have led to the death of at least 2.8 million individuals worldwide [1]. Moreover, 35% of Korean adults aged ≥18 years are reported to be obese [2].

Obesity is defined as abnormal or excess accumulation of fat that endangers health [1]. Although the Body Mass Index (BMI) is widely used to classify overweight and obesity, there are some caveats when using this value to determine excess body fat, the main one being that BMI does not discriminate between lean body mass and fat mass. Therefore, BMI can overestimate body fat in muscular athletes and underestimate sarcopenia in older adults [3]. Moreover, the cardiometabolic risks of obesity and their heterogeneity cannot be assessed by BMI alone [4].

Obesity is a consequence of multiple genetic, socioeconomic, lifestyle, and environmental factors. However, the main driver of the obesity pandemic is likely to be changes in the global food system and accompanying changes in dietary behaviour [5, 6]. Several studies have shown a close link between obesity and dietary behaviour. Particularly snacking in the absence of hunger [7], consumption of fast foods [8], binge eating or food addiction [9], vulnerability to external food cues [10], preference for foods high in calories, fat, sugar, and salt, and frequently eating outside the home [11].

It is important to recognise the complex factors that shape and influence dietary behaviour, which can be classified as food choice, eating behaviour, and dietary/nutritional intake [12]. Food choice includes behaviours and other factors that precede food intake, such as preferences, frequency of purchase, food preparation, and intentions. Eating behaviour is categorised as eating habits, eating occasions, portion size, dieting, symptoms of disordered eating, and ‘pickiness’. Dietary/nutritional intake includes the specifics of what is consumed, such as dietary pattern, meal pattern, food intake, and food components.

Awareness of the different types of dietary behaviour has prompted a recommendation for a tailored approach to the treatment of obesity [13]. Conceptualising and classifying subtypes of obese individuals according to their dietary behaviour might allow more personalised and effective behavioural and nutritional treatments.

Latent class analysis (LCA) has been widely used to classify dietary behaviours associated with obesity [14–16]. It can also be used to divide a population into mutually exclusive subgroups and classify them exhaustively based on the intersection of multiple observed characteristics [17].

## Methods

### Aims, study design, and participants

The aims of this study were to classify the dietary behaviour of overweight or obese individuals into subgroups using LCA and to explore the relationship between each of these subgroups and cardiometabolic risk factors. This cross-sectional study was based on a retrospective review of the medical records of 259 patients who visited an outpatient weight management clinic at a tertiary hospital and underwent a dietary behaviour assessment between January 2014 and February 2019. Patients who were younger than 18 years, those for whom height or weight data were missing, and those with no dietary behaviour assessment records were excluded. The study protocol was approved in advance of enrolling patients by the Institutional Review Board at Seoul National University Bundang Hospital (IRB no. B-1904–532-114). The need for informed consent was waived in view of the retrospective nature of the study and the anonymity of the data.

### Sociodemographic and clinical variables

Our outpatient weight management clinic is attended by obese patients who want to lose weight or are referred by other departments. Patients who attend this clinic initially complete a self-administered questionnaire designed to collect information on sociodemographic and lifestyle factors, a weight history, and eating-related behaviours, and then attend a consultation with a family physician specialised in obesity management. At the next session, a dietician performs a dietary assessment, provides the patient with detailed education on nutrition, and prescribes a low or very low calorie diet. Next, the patient attends an appointment with a doctor who checks for adherence or barriers to the prescribed diet or the exercise recommendations and, if needed, prescribes appetite-suppressing medication. These sessions are repeated at intervals of 2 weeks for 1 month, 4 weeks for 2 months, and 8 weeks for 4 months until the patient has achieved a weight decrease of at least 10% of the initial body weight. Patients are then encouraged to attend a weight loss maintenance program, where they learn strategies for maintaining the weight lost, and regular follow-up sessions for at least 12 months.

In this study, we collected sociodemographic information on sex, age, income, and level of education from the self-completed questionnaires. Lifestyle factors, such as smoking, hazardous drinking, and frequency of exercise, were included. Anthropometric measurements, including height, weight, systolic and diastolic blood pressure, and body fat were obtained by bioelectrical impedance analysis using the InBody 720 device (BioSpace Inc., Urbandale, IA, USA). Clinical variables included total cholesterol, high-density and low-density lipoprotein cholesterol (HDL and LDL cholesterol), triglycerides (TG), fasting blood glucose (FBS), glycated haemoglobin (HbA_1C_), estimated glomerular filtration rate and liver function tests including aspartate transaminase (AST), alanine transaminase (ALT) and gamma-glutamyl transferase (GGT).

Since mood disorders such as depression might affect eating behaviours of study subjects, we extracted the patient’s diagnosis of depression by using the tenth revision of the International Statistical Classification of Diseases and Related Health Problems (ICD-10). An ICD-10 diagnosis of F20.4, F31.3-F31.5, F32.x, F33.x, F34.1, F41.2, or F43.2 and two depression prescriptions given within a 1-year period were considered proxies for having depression [18]. Diagnosis was extracted using electronic medical records for a period of approximately 2 years from the day the questionnaire was conducted.

Using the National Cholesterol Education Program—Adult Treatment Panel III criteria for Asian populations [19], a diagnosis of metabolic syndrome was made when at least three of the following five conditions were met:

- High blood pressure (systolic ≥130 mmHg or diastolic ≥85 mmHg) or on antihypertensive medication
- High FBS level (≥100 mg/dL) or current use of oral antidiabetic medication
- High TG level (≥150 mg/dL) or current use of lipid-lowering medication
- HDL cholesterol (< 40 mg/dL in men, < 50 mg/dL in women)
- Abdominal obesity (≥90 cm in men, ≥85 cm in women)

### Dietary behaviour assessment

The self-administered dietary behaviour questionnaire included the following items, each of which was rated on a 5-point Likert scale: frequency of meals, skipping meals, frequent snacking or night eating, overeating or binge eating, preference for fatty foods or carbohydrates, consumption of instant food such as ramen, fast foods such as pizza or hamburgers, greasy food such as fried chicken, intake of sugar-sweetened beverages, and frequency of intake of carbohydrates, vegetables and fruit, protein, fat and dairy products; Table 1). Based on previous studies [20–23], we organised our dietary behaviour questions into three main domains with the following nine categories: food choice (frequently eating out, consumption of fast food, instant food, and takeaway food), eating behaviour (irregular meals, frequent snacking including at night, emotional eating, and overeating/binge eating), and nutritional intake (high-fat/high-calorie foods, salty food, and poorly balanced food intake).

**Table 1:** Dietary behaviour questionnaires

Category	Dietary behaviour	Question
Food choice	Frequently eating out	Do you usually eat out (except staff restaurant) about twice a week?
		Do you overeat when you eat out (except in the staff cafeteria)?
	Fast/instant/takeaway food	Do you eat bread, pizza, and chicken instead of your regular meal at dinner?
		Do you often eat instant food or fast food such as ramen?
Eating behaviour	Meal irregularity and speed	How often do you skip meal?
		Do you eat at a regular time?
		How fast do you eat your meal?
	Frequent or night snacking	Do you eat snacks within 3 h before sleep?
		Do you eat your dinner late at night?
		Do you sometimes eat snacks when you are watching TV or reading the newspaper?
	Overeating/binge eating	Do you eat more food than you had planned to?
		Can you stop eating when you are full?
		Do you continue to eat even after you feel like stopping?
		Do you overeat frequently?
		Do you sometimes eat food although you are not hungry?
	Emotional eating	Do you eat food on impulse?
		Do you eat food when you feel depressed or unhappy?
		Do you eat food when you feel anxiety or stress?
		Do you feel that you can recover yourself through eating sugary food?
Nutrient	High-calorie dense food	Do you eat foods containing sugar (crackers, candy, chocolates, cakes, ice cream) every day?
		Do you drink soft drinks, vitamin drinks, or fruit juice instead of water
		Do you often eat greasy food such as fried food, stir-fries, or salad dressing?
		Do you like meat?
	Salty food	Do you usually eat a lot of kimchi or salt-pickled vegetables?
		Do you eat all the broth in soups or stews?
		Do you often add salt or soy sauce before eating soups?
	Unbalanced food	Are you having fruits or fruit juice every day?
		Do you eat vegetables in each meal (except kimchi)?
		Are you eating at least one of the following foods: meat, fish, egg, bean, and tofu when you have a meal?
		Do you prefer to eat multi-grain rice over white rice?

We reclassified the food choice and eating behaviour domains as dichotomous variables and recorded ‘yes’ if items in those categories were responded to as ‘frequently’ or ‘very frequently’. For nutritional balance, we referenced the nutrition quotient calculation for Korean adults [24]. Briefly, the Korean nutrition quotient consists of nutrition balance, food diversity, moderation of amount of food intake, and dietary behaviour. Nutritional balance assesses the intake of vegetable/fruit, protein (fish, eggs, and beans), nuts and milk, and dairy products. If the calculated nutrition balance score was in the lower quartile, food intake was classified as unbalanced.

High-fat/high-calorie intake included high consumption of fatty foods, such as fried food, meat, ham, or sausage, and high consumption of sugar-sweetened beverages or foods containing sugar. Each score was summed to yield a mean score. If the mean high-fat/high-calorie score was > 4, it was classified as ‘yes’, as for a dichotomous variable. Other latent variables were classified as ‘yes’ or ‘no’ according to the corresponding mean scores.

### Statistical methods

Continuous variables are presented as mean and standard deviation and categorical variables as the frequency count and percentage. LCA was performed using PROC LCA (version 1.3.2) [25–27] and was used to classify the patients into nine subtypes of dietary behaviour. The Akaike information criterion and Bayesian information criterion were used to find the best representative number of subgroups in these data. The number of classes was selected by a combination of parsimony and interpretability, and the class number can be possibly used as a meaningful label for each class [27]. Each class was described in terms of sociodemographic factors, anthropometric measurements, and laboratory results.

We examined the association between LCA-derived classes and cardiometabolic risk factors using the LCA distal outcome Macro program (http://methodology.psu.edu/downloads). Briefly, LCA with a distal outcome was constituted with a ‘three-step’ method (Bolck–Croon–Hagenaars method) [28], whereby the parameters of the LCA model are first estimated without the distal outcome, the posterior probabilities of class membership are then used to compute a weighting variable, and finally, the weighting variable is used to calculate a weighted average of outcomes for each class. The LCA-driven classes were subsequently entered into a logistic regression model to test for associations between each class (with the relatively healthy class chosen as the referent) and the risk of metabolic syndrome with adjustment for age, sex, healthy behaviours, BMI, and other clinical variables. All statistical analyses were performed using SAS version 9.3 (SAS Institute Inc., Cary, NC, USA).

## Results

LCA identified three classes of study subjects. The model fit indices are summarised in Table 2. LCA models with 2–9 classes were compared for model fit and interpretability. We chose a three-class model that showed the best fit measured by a lower Bayesian information criterion with higher entropy.

**Table 2:** Model-fit indices for the latent class analysis model

Number of classes	AIC	BIC	Likelihood ratio G^2^	Degrees of freedom	Entropy
2	341.7	409.3	303.7	492	0.70
3	322.5	425.6	264.5	482	0.73
4	313.3	452.0	235.3	472	0.72
5	315.8	490.1	217.8	462	0.75
6	318.7	528.5	200.7	452	0.78
7	325.8	571.3	187.8	442	0.82
8	334.8	615.8	176.8	432	0.80
9	343.7	660.3	165.7	422	0.83

**Fig. 1:**
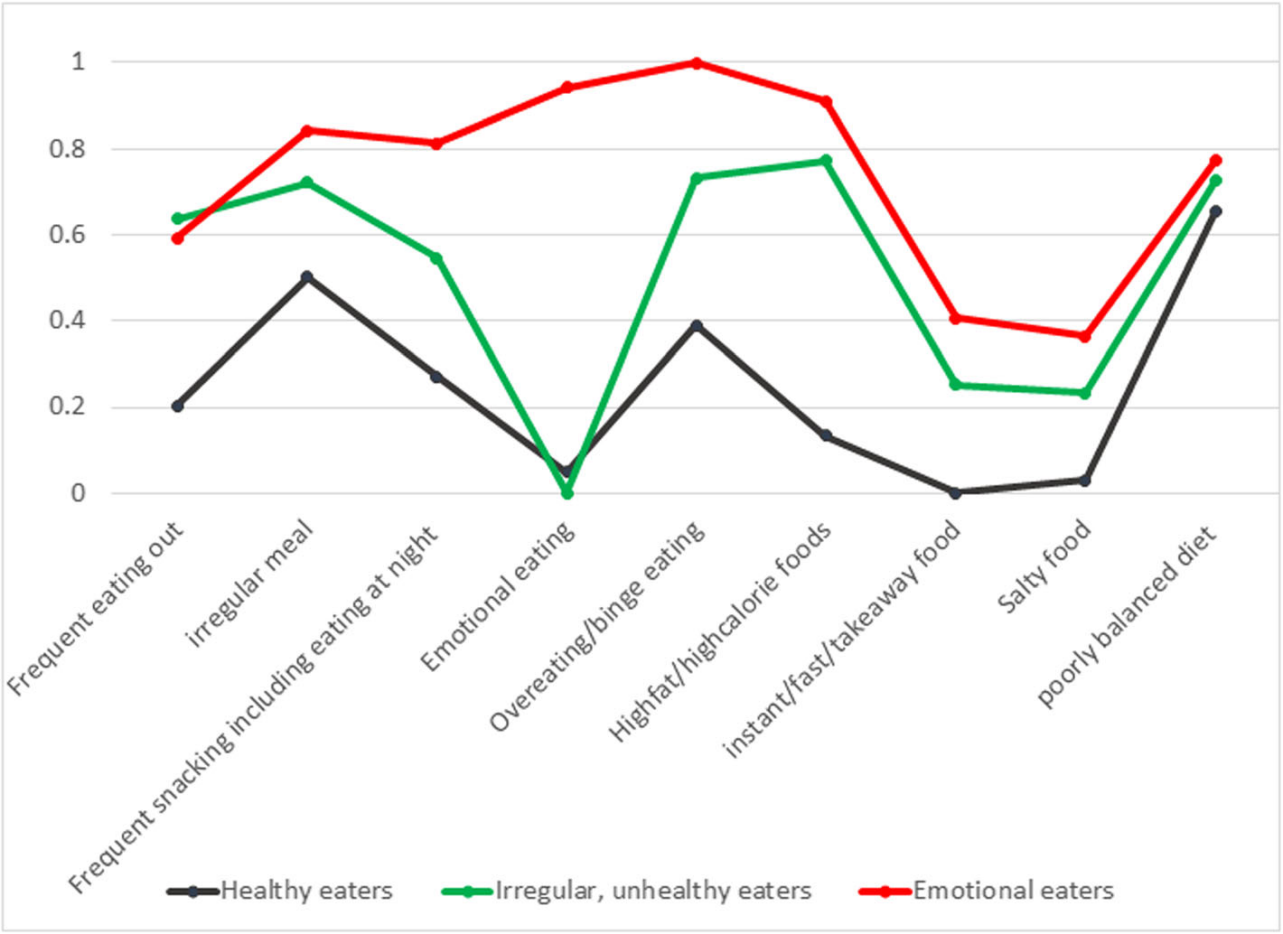
Item probabilities in the three latent classe

Figure 1 shows the conditional probabilities for each dietary behaviour in each class. Class 1 subjects (healthy but unbalanced eaters, *n* = 118) generally had regular meals and fewer snacks, reported less emotional eating, and did not eat fast/instant/takeaway food but tended to have a poorly balanced food intake. Class 2 subjects (irregular, unhealthy eaters, *n* = 88) were characterised by frequently eating out, irregular meal intake, a preference for calorie-dense food, and poorly balanced food intake, but no emotional eating. Class 3 (emotional eaters, *n* = 53) had a very high probability of emotional eating, binge eating, preference for calorie-dense food, irregular meals, and frequent eating at night.

Table 3 summarises the sociodemographic and clinical factors and the health-related behaviours in each class. Subjects in the emotional eating group were younger, had a higher BMI, and were more likely to be female than those in the other two groups. Furthermore, the prevalence of metabolic syndrome was 49% in the emotional eating group and 32% in both the healthy eating and irregular unhealthy eating groups. The emotional eaters were more likely to exercise regularly and less likely to smoke or consume alcohol than the other two groups.

**Table 3:** Baseline characteristics according to LCA-derived classes

	Class 1	Class 2	Class 3
	Healthy but unbalanced eaters	Irregular, unhealthy eaters	Emotional eaters
n	118	88	53
Age, years	50.8 (10.0)	46.5 (9.8)	42.6 (9.7)
Sex			
Male	76 (64)	65 (74)	22 (42)
Female	42 (36)	23 (26)	31 (58)
Underlying disease			
Hypertension	38 (42)	33 (65)	10 (28)
Diabetes	16 (20)	9 (18)	4 (18)
Hyperlipidaemia	28 (33)	29 (45)	15 (47)
Depression	13 (11)	13 (15)	7 (13)
Body Mass Index, kg/m^2^	27.2 (3.5)	28.0 (3.5)	30.7 (4.1)
Body fat percentage	29.5 (6.6)	28.9 (6.5)	34.8 (7.2)
Waist circumference, cm	93.8 (8.5)	95.2 (8.4)	99.5 (10.0)
Systolic blood pressure, mmHg	119.3 (14.2)	120.8 (15.4)	124.1 (14.9)
Diastolic blood pressure, mmHg	71.6 (10.0)	72.5 (11.5)	72.9 (8.9)
Education, n (%)			
High school	52 (44)	33 (38)	18 (34)
More than college	66 (56)	55 (63)	35 (66)
Income per month, n (%)			
Less than 8000 USD	42 (38)	20 (25)	8 (17)
More than 8000 USD	70 (63)	60 (75)	39 (83)
Smoking, n (%)			
No	46 (43)	29 (37)	28 (65)
Ex	35 (33)	24 (31)	7 (16)
Yes	26 (24)	25 (32)	8 (19)
Drinking, n (%)			
No	37 (32)	27 (31)	23 (45)
Yes	77 (68)	59 (69)	28 (55)
Exercise, n (%)			
Regular	75 (69)	60 (71)	45 (87)
None	34 (31)	24 (29)	7 (13)
Clinical laboratory findings			
FBS, mg/dL	102.4 (26.8)	102.0 (22.7)	100.4 (35.0)
HbA1c, %	5.9 (0.9)	5.9 (0.9)	5.9 (1.1)
Total cholesterol, mg/dL	201.3 (38.9)	194.2 (35.0)	198.6 (45.0)
TG, mg/dL	153.0 (100.4)	158.2 (96.0)	152.1 (111.6)
HDL cholesterol, mg/dL	50.8 (13.2)	49.3 (10.4)	49.2 (11.3)
LDL cholesterol, mg/dL	124.3 (32.1)	118.4 (30.3)	122.3 (36.0)
eGFR, mL/min	94.7 (17.5)	97.9 (18.9)	105.3 (19.5)
AST, mg/dL	33.2 (26.8)	35.1 (38.8)	29.4 (14.1)
ALT, mg/dL	40.9 (35.8)	48.8 (52.4)	41.7 (27.9)
GGT, mg/dL	47.4 (42.5)	59.0 (50.2)	41.1 (25.4)
Metabolic syndrome, n (%)	37 (32)	25 (32)	21 (49)

*AST* aspartate transaminase, *ALT* alanine transaminase, *eGFR* estimated glomerular filtration rate, *FBS* fasting blood glucose, *GGT* gamma glutamyl transferase, *HbA*_*1C*_ glycated haemoglobin, *HDL* high-density lipoprotein, *LDL* low-density lipoprotein, *TG* triglycerides

Table 4 shows the association between latent class and cardiometabolic risk factors. There were significant differences in BMI, waist circumference, and body fat percentage between the three classes. BMI, waist circumference, and body fat percentage values were lower in class 1 (the healthy referent group) than in classes 2 and 3, with no differences between classes 2 and class 3. No significant differences were found in the cardiometabolic variables between the three classes, except for GGT, which was higher in class 2 than in class 1. The prevalence of metabolic syndrome was higher in class 3 than in class 1.

**Table 4:** Association between latent classes and cardiometabolic risk factors

Difference in means	Estimate	SE	Wald statistic	*P*-value
Body mass index				
Class 1 vs. class 2	2.70	0.77	12.3	< 0.001
Class 1 vs. class 3	3.40	069	23.9	< 0.001
Class 2 vs. class 3	0.71	0.67	1.12	0.290
Omnibus test			24.56	< 0.001
Waist circumference				
Class 1 vs. class 2	4.09	1.94	4.42	0.035
Class 1 vs. class 3	5.34	1.76	9.15	< 0.01
Class 2 vs. class 3	1.26	1.62	0.60	0.437
Omnibus test			9.24	< 0.01
Body fat percentage				
Class 1 vs. class 2	6.96	1.38	25.29	< 0.001
Class 1 vs. class 3	5.26	1.26	17.30	< 0.001
Class 2 vs. class 3	−1.70	1.21	1.99	0.158
Omnibus test			27.48	< 0.001
Systolic blood pressure				
Class 1 vs. class 2	3.49	3.28	1.13	0.287
Class 1 vs. class 3	5.04	2.76	3.34	0.0676
Class 2 vs. class 3	1.55	2.90	0.29	0.593
Omnibus test			3.35	0.187
Diastolic blood pressure				
Class 1 vs. class 2	0.63	2.16	0.08	0.768
Class 1 vs. class 3	1.43	1.75	0.67	0.411
Class 2 vs. class 3	0.80	2.11	0.14	0.703
Omnibus test			0.68	0.712
TG				
Class 1 vs. class 2	−5.11	22.18	0.05	0.817
Class 1 vs. class 3	−0.73	19.67	0.00	0.970
Class 2 vs. class 3	4.37	18.67	0.05	0.814
Omnibus test			0.07	0.965
HDL cholesterol				
Class 1 vs. class 2	1.09	2.23	0.23	0.626
Class 1 vs. class 3	−1.88	2.17	0.75	0.384
Class 2 vs. class 3	−2.97	2.14	1.92	0.165
Omnibus test			1.98	0.370
FBS				
Class 1 vs. class 2	−2.37	6.66	0.13	0.721
Class 1 vs. class 3	−1.25	6.06	0.04	0.836
Class 2 vs. class 3	1.12	4.83	0.05	0.816
Omnibus test			0.13	0.936
GGT				
Class 1 vs. class 2	−19.4	7.29	7.06	< 0.01
Class 1 vs. class 3	−7.14	6.38	1.25	0.262
Class 2 vs. class 3	12.25	8.70	1.98	0.158
Omnibus test			7.44	0.024
Metabolic syndrome (difference in log means)				
Class 1 vs. class 2	−0.76	0.46	2.67	0.102
Class 1 vs. class 3	0.82	0.39	4.26	0.038
Class 2 vs. class 3	0.05	0.43	0.01	0.902
Omnibus test			4.73	0.093

*FBS* fasting blood glucose, *GGT* gamma glutamyl transferase, *HDL* high-density lipoprotein, *TG* triglycerides

Logistic regression was performed to evaluate the associations between the three LCA-driven classes and metabolic syndrome using healthy but unbalanced eaters as the referent group (Table 5). Significant association of metabolic syndrome with emotional eating, but not with irregular unhealthy eating (class 2), was found when compared with the referent group (class 1). Logistic regression analysis showed that metabolic syndrome was associated with a higher BMI, an increase in liver enzyme levels (> 120 mg/dL, equivalent to three times the upper limit of normal), and current smoking.

**Table 5:** Odds ratio and 95% confidence interval for metabolic syndrome in association with latent classes and covariates

Variable	Odds ratio	95% confidence interval	*P*-value
Class 1: healthy but unbalanced eaters	Ref		
Class 2: irregular, unhealthy eaters	1.12	0.54, 2.34	0.761
Class 3: emotional eaters	2.88	1.16, 7.13	0.012
Age 20–39 years	Ref		
Age 40–59 years	1.04	0.31, 3.54	0.946
Age > 60 years	1.37	0.34, 5.45	0.657
Male	Ref		
Female	0.94	0.35, 2.55	0.903
BMI 23–25 kg/m^2^	Ref		
BMI > 25 kg/m^2^	2.62	1.00, 6.89	0.051
Liver enzymes < 40 mg/dL	Ref		
Liver enzymes 40–120 mg/dL	1.81	0.89, 3.73	0.108
Liver enzymes > 120 mg/dL	5.21	1.47, 18.52	0.010
Non-smoker	Ref		
Ex-smoker	0.94	0.33, 2.64	0.901
Current smoker	3.03	1.04, 8.83	0.042
No drinking	Ref		
Drinking	0.58	0.25, 1.32	0.193
Regular exercise	Ref		
No exercise	1.35	0.65, 2.79	0.415

## Discussion

In this study, subtyping of dietary behaviour in overweight or obese individuals identified three latent classes (healthy but unbalanced eaters, irregular unhealthy eaters, and emotional eaters). Moreover, we identified a significant association between emotional eating and a higher BMI, waist circumference, and body fat percentage. Importantly, emotional eaters also had a higher prevalence of metabolic syndrome (emotional eaters: 49%, other eaters: 32%), which means that emotional eating can be a risk factor for metabolic syndrome. Recently, another longitudinal study using the data of the Korean Healthy Twin study [29] showed that baseline emotional eating behaviour had strong association with concurrent, incident, and persistent metabolic syndrome, which was compatible with our study except that the previous study’s results applied only to women. Emotional eating can be defined as eating in response to negative emotions or stress and is one of the many causes for weight gain or regaining weight after dieting [30]. It is known that stress and negative emotions can lead to a higher intake of palatable energy-dense foods, such as chocolate, cakes, biscuits, pizzas, hamburgers, French fries, and sausages [31]. Emotional eating might lead to increased intake of energy-dense foods and may induce weight gain, obesity, and metabolic syndrome. Patients with obesity already have a higher set point driven by hormonal balance and emotional eating in response to stress, which is governed by the reward system of the brain that can override the metabolic set point, leading to sustained weight gain above the metabolic set point [32]. In our study, the emotional eater group might have been driven by hedonic system over metabolic system of body weight regulation, which can lead to higher BMI and associated metabolic syndrome.

Despite their different dietary profiles, subjects in classes 1 and 2 showed no significant differences in cardiometabolic risk factors except for GGT. This might reflect similar distributions of age, sex, and underlying disease. Irregular unhealthy eaters were more likely to have irregular meals, dine out, and consume alcohol (which might explain the increased GGT in this group). In contrast, classes 2 and 3 had similar dietary behaviour patterns, except for emotional eating, which might have contributed to their higher rates of binge eating, irregular meals, and frequent snacking, which in turn could lead a higher BMI and a higher likelihood of metabolic syndrome.

In a study performed in the Netherlands, emotional eating was found to be a mediator between depression and 5-year weight gain in mothers [33]. In that study, depressive symptoms were associated with higher rates of emotional eating, which resulted in an increase in BMI independent of depressive symptoms. Emotional eating is also closely associated with insufficient sleep and poor sleep quality [30]. Insufficient sleep can cause more negative emotions and interferes with regulation of emotion through neurobiological, cognitive, and behavioural pathways. Therefore, identifying the subtype of obesity, such as emotional eating, can facilitate tailored treatment, such as training in emotional regulation skills or improving other lifestyle factors such as sleep.

The cross-sectional nature of this study meant that we could not detect a difference in the baseline cardiometabolic risk factors between subjects with healthy dietary behaviours and those with unhealthy behaviours (classes 1 and 2). However, it was worthwhile to subtype the group with irregular unhealthy dietary behaviour for the purposes of prediction and intervention. Several studies have suggested that an unhealthy dietary pattern can lead to increased weight, BMI, and waist circumference [34, 35] and that this group of patients might achieve greater weight loss if they receive more tailored interventions targeting multiple health behaviours rather than strategies that target a single behaviour [36].

To our knowledge, this is the first study in Korea that has used LCA to classify dietary behaviour and explore its association with metabolic syndrome in overweight or obese adults. Dietary behaviours are highly inter-related, often concurrent, and affected by a complex interplay of multiple risk factors, including socioeconomic status and other health-related behaviours. LCA can address the complexity of dietary behaviour and capture meaningful key patterns [27].

Logistic regression analysis of metabolic syndrome and LCA-derived emotional eating showed significant associations but not when irregular unhealthy eaters were compared with healthy but unbalanced eaters, indicating that some components of dietary behaviour have a greater effect than others.

The findings of this study suggest a practical approach for identifying different phenotypes amongst individuals who are overweight or obese. Emotional eaters should be prioritised for emotional regulation and encouragement of emotional well-being. A recent review suggested that mindfulness and meditation have the potential to decrease emotional eating [37].

According to the American Heart Association, the timing and frequency of meals is also important in the management of cardiometabolic risk factors [38]. Regular meals with avoidance of late-night snacking can attenuate the risk of heart disease and diabetes mellitus. In this regard, identifying individuals with unhealthy dietary behaviours, such as irregular mealtimes or frequent snacking (class 2), and managing them with the focus on eating patterns might help achieve a healthy cardiometabolic profile as well as effective weight reduction.

This study has several limitations. First, it was not possible to use a validated dietary questionnaire because the study was based on a retrospective review of medical charts. Second, categorising the components of the dietary behaviour questionnaire into nine items could be considered subjective; however, we attempted to offset this problem by referencing a dietary pattern evaluation tool devised for Koreans [23]. Third, the questionnaire used in the study was self-administered, which might have introduced a degree of reporting bias. Fourth, the study data were obtained from a single hospital weight management clinic and might not be generalisable to other populations. Dietary behaviours can be influenced by sex, age, and socioeconomic factors [39, 40]. Neither sex-specific nor age-specific LCA could be performed in this study because of the relatively small number of patients enrolled after exclusion of those with missing data. Lastly, since depression seemed strongly associated with emotional eaters, evaluating depressive symptoms were important tools in understanding emotional eaters compared with other types of eaters. However, no validated questionnaires were available regarding depression, we used operational definition of depression using the ICD-10 codes and prescription [18]. Although it has a high specificity (94.3%), its moderate sensitivity (61.4%) might limit the exact status of depression amongst the study subjects. Further detailed methods for detecting depression or sleep problem will be required using validated questionnaires relevant to eating disorder or eating behaviours.

Despite its drawbacks, this study shows that subtyping obesity-related dietary behaviours could be a guide to prioritising the components that should be put in place for tailored cognitive behavioural therapy. Further rigorous research is needed for these interventions to be effective in weight management.

## Conclusions

In conclusion, we have shown that LCA-driven obesity phenotyping can be a useful tool for assessment and management of obesity as well as metabolic syndrome. In this study, emotional eaters had higher BMI and were more likely to have metabolic syndrome than other types of eaters. Emotional eaters might benefit from strategies targeting emotional regulation. People with irregular unhealthy dietary behaviours, such as irregular mealtimes or frequent night snacking, could be candidates for cognitive behavioural therapy focusing on healthy eating behaviours, which can also contribute to a favourable cardiometabolic profile.

## Data Availability

The datasets used and analysed during the current study are available from the corresponding author on reasonable request.

## Abbreviations

ALT: Alanine transaminase
AST: Aspartate transaminase
BMI: Body Mass Index
eGFR: Estimated glomerular filtration rate
FBS: Fasting blood glucose
GGT: Gamma glutamyl transferase
HbA_1C_: Glycated haemoglobin
HDL: High-density lipoprotein
LDL: Low-density lipoprotein
LCA: Latent class analysis
TG: Triglycerides
